# Efficacy of Extracorporeal Shock Wave and Pulse Electromagnetic Field Therapies in Calcaneal Spurs

**DOI:** 10.34172/aim.2023.94

**Published:** 2023-11-01

**Authors:** Gülşah Yaşa Öztürk, Ayşegül Yetişir

**Affiliations:** ^1^Department of Physical Medicine and Rehabilitation, Adana City Training and Research Hospital, Adana, Turkey; ^2^Çukurova University, Faculty of Medicine, Department of Physical Medicine and Rehabilitation, Division of Rheumatology, Adana, Turkey

**Keywords:** Calcaneal spur, Extracorporeal shock wave therapy, Foot function index, Pulse electromagnetic field therapy, Visual analogue scale

## Abstract

**Background:**

Various treatment methods are available for calcaneal spur, which can cause disability.

**Objective:**

To evaluate the efficacy of pulsed electromagnetic field therapy (PEMFT) added to extracorporeal shock wave therapy (ESWT) on pain and functional capacity in treating calcaneal spurs.

**Methods:**

Patients with calcaneal spurs who were recommended ESWT or ESWT+PEMFT and whose Foot Function Index (FFI) and visual analogue scale (VAS) values were available in their records were retrospectively analyzed. The two groups were ESWT (n=35) and ESWT+PEMFT (n=40). FFI and VAS scores were obtained from their records before treatment, after treatment, and in the third month after treatment.

**Results:**

The two groups were similar regarding their pre-treatment FFI and VAS scores. In intra-group evaluation, statistically significant decreases were found in terms of the FFI pain, disability, and activity limitation and VAS scores in both groups after treatment and in the third month after treatment compared to the pre-treatment period. In the comparison between the groups, the post-treatment and post-treatment third-month FFI pain, disability, and activity limitation and VAS scores were significantly lower in the PEMFT+ESWT group than the ESWT group (*P*<0.001).

**Conclusion:**

A calcaneal spur is a condition that can cause pain and functional limitation in patients. Various studies have demonstrated the efficacy of ESWT in the treatment of calcaneal spurs. In our study, we observed that PEMFT added to ESWT significantly improved the pain and functionality of the patients. Further studies are needed to evaluate the efficacy of PEMFT in calcaneal spurs.

## Introduction

 A calcaneal spur, which can cause severe pain in the heel region and limit activities of daily living, has a prevalence of 11%‒21% in the young and middle-aged population; in those over the age of 62 years, this rate rises to 55%. Repetitive stress and traction on the insertion area of the plantar fascia and the intrinsic musculature in the calcaneus cause inflammation, ultimately leading to the development of a spur in that area. This results in tenderness in the heel, with maximum tenderness found in the area of the spur.^1^ Patients usually present with pain in the anteromedial aspect of the calcaneus. Pain worsens upon standing after a period of rest, and it is typical when the first step is taken in the morning.^2^ A calcaneal spur is detected on lateral foot direct radiography.^3^ Since it is a condition that causes disability, various treatment options are applied. Conservative and interventional methods include lifestyle modification, night splints, calcaneal cups, stretching exercises, physical therapy modalities, extracorporeal shock wave therapy (ESWT), steroids, platelet-rich plasma injections, and surgery.^2^

 Shockwave therapy is widely used in the treatment of tendon injuries, and there is increasing evidence for its clinical efficacy.^4^ Strong shock waves break up the scar tissue, stimulate angiogenesis, stimulate new bone formation, fragment calcific deposits, and increase cytokine diffusion.^5^ Recent systematic reviews and meta-analyses have shown that ESWT is an effective treatment with success rates reported between 50% and 94%.^4^

 Another physical therapy modality is pulsed electromagnetic field therapy (PEMFT), which uses a time-varying magnetic field created by an electric current passing through a conductor.^6^ It transmits electromagnetic energy to the soft tissue, creating a therapeutic effect. Its analgesic effects have been shown in neck pain, osteoarthritis, and postoperative pain.^7^ In animal models, increased fracture healing and collagen sequencing, decreased inflammation, and tissue healing have been demonstrated.^8-10^ In light of this information, we aimed to investigate the efficacy of PEMFT in the treatment of calcaneal spurs as a contributor to the proven effect of ESWT.

## Materials and Methods

 Patients aged 18‒75 years, who presented to our outpatient clinic between January 1, 2022, and June 1, 2022, and were diagnosed with calcaneal spurs, were retrospectively analyzed.We found 90 patients who received ESWT (group 1) and 65 patients who received ESWT and PEMFT (group 2) between 01.01.2022 and 01.06.2022. Seventy patients in the ESWT group and 55 patients in the ESWT and PEMFT group met the inclusion criteria. Power = 80%, confidence interval = 95%, d = 0.5 taken as the two-tail test, and the minimum number to be recruited as the sample size was 35 patients in group 1 and 40 patients in group 2. Finally, 35 patients were included in the ESWT group and 40 patients were included in the ESWT and PEMFT group by simple randomization in this study. Randomization was done by an individual not involved with the study. All patients included in the study had a visual analogue scale (VAS) score above 3 in the subcalcaneal region for ≥ 1 month, were recommended ESWT or ESWT + PEMFT, and had their pre-treatment and follow-up data on the Foot Function Index (FFI) and VAS available in their records. The pre-treatment, post-treatment, and post-treatment third-month scores ​​were examined.

 The exclusion criteria were as follows: having received any physical therapy within the last six months or having used analgesics or antimuscarinic agents within the last week, presence of peripheral vascular disease, type 2 diabetes mellitus (DM), osteoporosis, acute trauma to the foot, fracture or surgery history, lower extremity neurological deficit, polyneuropathy, lumbar pathology findings that could cause foot pain, rheumatological disease, history of anticoagulant use, tumors, thrombosis, soft tissue or bone infection, acute inflammation, epilepsy, hematological disease, or coagulation disorder, hemoglobin level < 11 g/dL, platelet count < 150 000/mm^3^, pregnancy, breastfeeding, having a pacemaker, and presence of skin lesions in the application area.

 In the VAS evaluation, each patient was asked to mark the severity of pain on a 100 mm line, with “no pain” at one end and “most unbearable pain” at the other end, and the result was recorded. FFI was originally developed to assess foot pain, disability, and activity limitation, and the validity and reliability analyses of the Turkish version were confirmed by Anaforoğlu Külünkoğlu et al.^11^ FFI consists of 23 items (nine for pain, nine for disability, and five for activity limitation). Each item is scored on a 10-point scale.

 As part of routine practice in our clinic, ESWT (Roland Health, Elettronica Pagani) (6.0 Hz, 500 shock waves, 1.7 bar pressure) is applied to painful areas in the calcaneal area for a total of five sessions at four-day intervals to some patients diagnosed with calcaneal spurs. PEMFT is added to the treatment of some other patients depending on their clinical state and physician’s availability. Before ESWT application, magnetic field therapy is routinely applied to the area covering the calcaneus at a dose of 20G and low frequency of 10-100 Hz (Roland Health, Elettronica Pagani) for 20 minutes (five sessions in total at four-day intervals).

###  Statistical Analysis

 The Statistical Package for the Social Sciences v. 23.0 package program was used for statistical analysis of the data. Categorical measurements were summarized as numbers and percentages, and continuous measurements as mean and standard deviation (median and minimum-maximum where appropriate). The chi-square tests were used to compare categorical variables. The Shapiro-Wilk test was conducted to determine whether the variables included in the study showed a normal distribution. The Mann-Whitney U test was used for non-normally distributed variables. The differences between the patients’ pre-treatment, post-treatment, and post-treatment third-month findings were evaluated with the repeated-measures analysis, and the Wilcoxon rank test was performed to analyze the findings that were significant. The statistical significance level was taken as 0.05 for all the tests.

## Results

 Seventy-five patients diagnosed with a calcaneal spur were included in the study. Group 1 (n = 35) received only ESWT, while group 2 (n = 40) received PEMFT in addition to ESWT. Table 1 presents the data on gender, age, body mass index, additional disease, spur side, and spur size measured on direct radiographs according to the study groups. Comorbidities (DM and hypertension) were observed in a total of 14 patients (18.7%).

**Table 1:** Gender, Comorbidities, Spur Direction, Age, BMI, and Spur Size of the Study Groups

	**ESWT ** **(n=35)**	**ESWT+PEMFT** **(n=40)**	***P*** ** Value**
Gender, No. (%)			0.200^a^
Female	29 (83)	37 (93)	
Male	6 (17)	3 (7)	
Presence of comorbidity, No. (%)	5 (14)	9 (23)	0.362^a^
Spur side, No. (%)			0.279^a^
Right	14 (40)	21 (53)	
Left	21 (60)	19 (47)	
Age (y), Median (IQR)	50 (13)	52 (3.5)	0.081^b^
BMI (kg/m^2^), Median (IQR)	31 (3.9)	31 (3.94)	0.369^b^
Spur size (mm), Median (IQR)	5 (2.8)	5 (2.9)	0.227^b^

ESWT, Extracorporeal shock wave therapy; PEMFT, Pulse electromagnetic field therapy; BMI, body mass index. ^a^Chi-square test; ^b^ Mann-Whitney U test.

Table 2 shows the changes in groups 1 and 2 over the evaluation period (from pre-treatment to post-treatment and from pre-treatment to post-treatment third month).

**Table 2:** Comparisons of Changes in the FFI and VAS Scores of the Groups According to the Evaluation Time

	**Pre-treatment**	**Post-treatment**	**Post-treatment Third Month**	***P***	***P*** **1**	***P*** **2**	***P*** **3**
	**Median (IQR)**	**Median (IQR)**	**Median (IQR)**				
ESWT							
FFI pain	81 (13)	65 (32)	65 (36)	< 0.001**	< 0.001**	< 0.001**	0.943
FFI disability	80 (15)	60 (50)	40 (50)	< 0.001**	< 0.001**	< 0.001**	0.002*
FFI activity limitation	45 (6)	40 (26)	40 (27)	< 0.001**	< 0.001**	< 0.001**	0.492
VAS	9 (1)	7 (4)	7 (4)	< 0.001**	< 0.001**	< 0.001**	0.039*
ESWT + PEMFT							
FFI pain	86 (13.25)	12 (12)	11.5 (5)	< 0.001**	< 0.001**	< 0.001**	< 0.001**
FFI disability	84 (17)	13.5 (14.5)	11 (8.5)	< 0.001**	< 0.001**	< 0.001**	< 0.001**
FFI activity limitation	45 (7.75)	6 (9.5)	5 (5.75)	< 0.001**	< 0.001**	< 0.001**	< 0.001**
VAS	9 (0.75)	1 (1)	1 (1)	< 0.001**	< 0.001**	< 0.001**	0.005*

FFI, Foot Function Index; VAS, Visual Analog Scale; ESWT, extracorporeal shock wave therapy; PEMFT, pulse electromagnetic field therapy. *P < 0.05; **P < 0.001; *P*, repeated-measures ANOVA; *P*1, pre-treatment vs post-treatment; *P*2, pre-treatment vs post-treatment third month; *P*3, pre-treatment vs post-treatment third month; *P*1-3, Wilcoxon signed-rank test.

 The two groups were similar in terms of the pre-treatment FFI pain, disability, and activity limitation and VAS scores. Table 3 shows the comparison of the pre-treatment and post-treatment FFI and VAS scores of the patients.

**Table 3:** Comparison of the FFI and VAS Scores Between the Groups According to the Evaluation Time

		**ESWT (*****n*****=35)**	**ESWT+PEMFT (*****n*****=40)**	***P*****Value**^a^
		**Median (IQR)**	**Median (IQR)**	
FFI pain	Pre-treatment	81 (13)	86 (13.25)	0.187
	Post-treatment	65 (32)	12 (12)	< 0.001
	Post-treatment third month	65 (36)	11.5 (5)	< 0.001
	Pre-treatment	80 (15)	84 (17)	0.192
FFI disability	Post-treatment	60 (50)	13.5 (14.5)	< 0.001
	Post-treatment third month	40 (50)	11 (8.5)	< 0.001
	Pre-treatment	45 (6)	45 (7.75)	0.322
FFI activity limitation	Post-treatment	40 (26)	6 (9.5)	< 0.001
	Post-treatment third month	40 (27)	5 (5.75)	< 0.001
	Pre-treatment	9 (1)	9 (0.75)	0.096
VAS	Post-treatment	7 (4)	1 (1)	< 0.001
	Post-treatment third month	7 (4)	1 (1)	< 0.001

FFI, Foot Function Index; VAS, visual analog scale; ESWT, Extracorporeal shock wave therapy; PEMFT, Pulse electromagnetic field therapy, ^a^ Mann-Whitney U test.

## Discussion

 There are various ongoing studies on the effective treatment of calcaneal spurs, which can cause pain and decreased functional status in the general population. A multicenter, prospective, randomized, double-blind, placebo-controlled study evaluated the effects of using a wearable PEMFT device for seven nights in 70 patients (42 in the treatment group and 28 in the placebo group). Pain in the morning (with the first step taken) and pain in the evening (before sleeping) were evaluated. The patients were free to use pain medication. Morning pain in the active treatment group showed a significant decrease between day 1 and day 7 compared to the placebo group. In the active treatment group, the reduction in morning pain became significant on day 4 compared to day 1, and this significance remained until day 7. Evening pain decreased by 30% in the active treatment group and by 19% in the placebo group compared to the baseline, but the difference was not significant. Drug use also tended to decrease in the active treatment group, but it remained consistent with day 1 levels in the placebo group. The authors concluded that a wearable PEMFT device was a simple, drug-free, and non-invasive treatment option for heel pain.^7^ In another study, 29 patients with rotator cuff tendinitis were treated in placebo and PEMFT groups. On the completion of the study, the authors concluded that PEMFT might be useful in the treatment of severe and persistent rotator cuff tendinitis and possibly other chronic tendon lesions.^12^ In a study in which 60 patients with lateral epicondylitis were divided into three groups, it was determined that PEMFT sham reduced pain better than PEMFT.^13^ In another study, out of 53 patients with chronic Achilles tendinopathy, 28 were treated with a pulse electromagnetic field transduction therapy device (active treatment group) and 25 (control group) with a heel pad alone. There was a significant decrease in the VAS scores of the active treatment group compared to the control group. It was concluded that electromagnetic transduction therapy could be a potentially useful modality for the treatment of Achilles tendinopathy.^14^ Similar to ESWT, there are also studies indicating that mechanical stimulation using PEMFT may play a role in the treatment of tendinopathy and tendon regeneration by increasing *in vitro* TGF-β production and scleraxis and collagen I gene expression.^15^

 In the literature, we did not find any other study design similar to ours. When we compared the two groups of patients with calcaneal spurs, we observed that the ESWT + PEMFT group had a statistically more significant decrease in the post-treatment and post-treatment third-month FFI pain, disability, and activity limitation and VAS scores compared to the group that only received ESWT. In intra-group evaluation, the ESWT + PEMFT group had a significant decrease in the VAS and FFI pain, disability, and activity limitation scores in the pre-treatment versus post-treatment and pre-treatment versus post-treatment third-month comparisons.

 In a study on 80 patients with symptomatic calcaneal spurs, evaluation was made after two doses of ESWT. The pre-treatment and post-treatment third-month VAS scores were compared. The VAS score was found to be significantly lower after treatment.^16^ In another study, radial, focused, and sham ESWT treatments were applied to calcaneal spurs, and FFI scores were evaluated at the end of treatment and during the follow-up. Radial and focused ESWTs were found to be superior to the sham treatment.^17^ A systematic review and meta-analysis of the effects of ESWT on foot and ankle diseases identified 24 clinical studies and concluded that ESWT could assist in the treatment of plantar fasciitis and calcaneal spurs. When changes in VAS scores before and after treatment were examined in plantar fasciitis, ESWT was found to be effective compared to the placebo/conservative treatment.^18^ There are many studies showing that ESWT is effective in the treatment of calcaneal spurs. In the current study, a statistically significant difference was observed in the group that received ESWT alone in terms of the FFI pain, disability, and activity limitation and VAS scores after treatment and at the third month after treatment compared to the pre-treatment evaluation.

## Conclusion

 A calcaneal spur is a common condition that negatively affects the quality of life. Many methods are used in its treatment. In our study, we found that the application of ESWT alone was effective, but the addition of PEMFT to ESWT resulted in a more significant improvement. In the literature, studies on the use of PEMFT in calcaneal spurs are very limited, and therefore further research is needed on this subject.
